# Contribution of omics to biopreservation: Toward food microbiome engineering

**DOI:** 10.3389/fmicb.2022.951182

**Published:** 2022-08-02

**Authors:** Frédéric Borges, Romain Briandet, Cécile Callon, Marie-Christine Champomier-Vergès, Souad Christieans, Sarah Chuzeville, Catherine Denis, Nathalie Desmasures, Marie-Hélène Desmonts, Carole Feurer, Françoise Leroi, Sabine Leroy, Jérôme Mounier, Delphine Passerini, Marie-France Pilet, Margot Schlusselhuber, Valérie Stahl, Caroline Strub, Régine Talon, Monique Zagorec

**Affiliations:** ^1^Université de Lorraine, LIBio, Nancy, France; ^2^Université Paris-Saclay, INRAE, AgroParisTech, Micalis Institute, Jouy-en-Josas, France; ^3^Université Clermont Auvergne, INRAE, VetAgro Sup, UMR 545 Fromage, Aurillac, France; ^4^ADIV, Clermont-Ferrand, France; ^5^ACTALIA, Pôle d’Expertise Analytique, Unité Microbiologie Laitière, La Roche sur Foron, France; ^6^ACTALIA, Sécurité des Aliments, Saint Lô, France; ^7^Normandie Univ, UNICAEN, UNIROUEN, ABTE, Caen, France; ^8^Aerial, Illkirch, France; ^9^IFIP, Institut de la Filière Porcine, Le Rheu, France; ^10^Ifremer, MASAE, Laboratoire EM3B, Nantes, France; ^11^Université Clermont Auvergne, INRAE, MEDIS, Clermont-Ferrand, France; ^12^Univ Brest, Laboratoire Universitaire de Biodiversité et Ecologie Microbienne, Plouzané, France; ^13^Oniris, INRAE, SECALIM, Nantes, France; ^14^Qualisud, Univ Montpellier, Avignon Université, CIRAD, Institut Agro, IRD, Université de La Réunion, Montpellier, France

**Keywords:** shelf life, food, safety, pathogen, spoilage, microbiome, fermentation, biopreservation

## Abstract

Biopreservation is a sustainable approach to improve food safety and maintain or extend food shelf life by using beneficial microorganisms or their metabolites. Over the past 20 years, omics techniques have revolutionised food microbiology including biopreservation. A range of methods including genomics, transcriptomics, proteomics, metabolomics and meta-omics derivatives have highlighted the potential of biopreservation to improve the microbial safety of various foods. This review shows how these approaches have contributed to the selection of biopreservation agents, to a better understanding of the mechanisms of action and of their efficiency and impact within the food ecosystem. It also presents the potential of combining omics with complementary approaches to take into account better the complexity of food microbiomes at multiple scales, from the cell to the community levels, and their spatial, physicochemical and microbiological heterogeneity. The latest advances in biopreservation through omics have emphasised the importance of considering food as a complex and dynamic microbiome that requires integrated engineering strategies to increase the rate of innovation production in order to meet the safety, environmental and economic challenges of the agri-food sector.

## Introduction

Foods of animal or plant origins are complex ecosystems, rich in nutrients, with physicochemical characteristics enabling microbial growth during processing and storage. These ecosystems are colonised by microbial communities that can include pathogenic or spoilage microorganisms but also beneficial ones. The consumption of food contaminated with pathogens is an important cause of morbidity and mortality worldwide. Every year, approximately 600 million people – 1 in 10 people – get sick from foodborne pathogens, 420,000 of whom die. Human damage caused by foodborne pathogens results in colossal economic losses amounting to USD 110 billion due to lost productivity and health expenses (World Health Organization, 2015). Spoilage organisms are responsible for colour, odour, texture, taste or packaging defects leading to inedible products. Food microbial contaminants (spoilage as well as pathogenic microorganisms) contribute to global food loss and waste. According to the Food and Agriculture Organization (FAO), food waste occurs during the retail and consumption stages while food loss occurs after harvest or slaughter until retail (FAO, 2019). The causes of food waste and loss are numerous but a significant part of food destruction linked to microbial contamination is due to non-compliance with pathogen-related regulations or spoilage. At the European Union level, 20% of total available food is lost or wasted, fruits and vegetables being the most impacted category (43.5% of the food group), ahead of meat and fish products (26.3%; Caldeira et al., 2019). Worldwide, one-third of food produced for human consumption, about 1.3 billion tonnes per year, is estimated to be lost or wasted along the food supply chain (HPLE, 2014), while about 12% of the world population suffers from hunger (Agriculture and Economic Development Analysis Division, 2013). Reducing food wastage is thus crucial not only for ethical reasons but also for economic reasons. Food loss and waste are responsible for direct costs of about USD one trillion every year, but hidden costs extend much further. Indeed, global costs (including environmental, social, and economic costs) are evaluated by the FAO to amount to USD 2.6 trillion per year (FAO, 2014).

In addition, as food waste is correlated with high greenhouse gas emissions, reducing the undesirable impact of microorganisms has become a major objective in a situation of climate emergency. In this context, microorganisms also offer potential levers of action and can represent real opportunities for resolving food safety issues (Cavicchioli et al., 2019). In the global food system, approaches based on the barrier properties of biological systems appear attractive because of their efficiency and sustainability. These approaches are grouped under various names including biosanitation, biocontrol, bioprotection, and biopreservation. In this review, we will refer to food biopreservation, which is based on the hurdle technology that consists in using microorganisms (often lactic acid bacteria) as protective cultures and/or their metabolites to optimise the microbiological quality and shelf life of food by ensuring safety or reducing food waste, as defined by Stiles (Stiles, 1996). The use of protective cultures is often considered as an alternative to chemical additives or as a replacement for certain ingredients. Therefore, biopreservation should also help to meet the strong expectations of consumers who want “healthier” and more “natural” foods, and contribute to nutritional recommendations aimed at reducing salts, sugars, and additives in foods.

The concept of biopreservation was inspired by food fermentation ancestrally used to preserve food, except that fermentation involves substantial transformation of the food matrix, which is usually not the intention when engineering biopreservation systems. Consistently, the intentional addition of microorganisms or their metabolites for specific preservation purposes was largely investigated on fermented food, i.e., dairy products (cheeses, yoghurt), bakery products, or fermented sausages. Nevertheless, biopreservation was successfully extended to non-fermented food such as seafood, raw meat and non-fermented plant products. For example, biopreservation of seafood as fresh fish fillets, smoked fish or cooked shrimps aimed at controlling spoilers or pathogens such as *Listeria monocytogenes, Staphylococcus aureus, Vibrio* and histamine-producing bacteria (for a recent review, see Rathod et al., 2022). Biopreservation of raw meat (lamb, pork, beef, or poultry) and processed meat (sausage, cooked ham) has also focused on the control of pathogenic and spoilage bacteria as well as extension of shelf life (Jones et al., 2010; Zagorec and Champomier-Vergès, 2017; Wang et al., 2019). Biopreservation of non-fermented plant products is essentially dedicated to fighting against spoilage microorganisms including yeasts, moulds, spore-forming bacteria (*Bacillus subtilis, Bacillus licheniformis*), and pathogenic bacteria such as *L. monocytogenes* (Leyva Salas et al., 2017).

Most species used for food biopreservation by the food industry are lactic acid bacteria belonging essentially to the genera *Lactococcus*, *Lactobacillus lato sensu* and *Carnobacterium*. One strain of the Gram-negative species *Hafnia alvei* is also available on the market for anti-*Escherichia coli* purposes (Callon et al., 2016; Frétin et al., 2020). These bacteria are derived from food microbiota and are therefore particularly well adapted to food matrices. Moreover, as these species have been studied for several decades, their use as protective cultures is considered safe. *Latilactobacillus sakei, Latilactobacillus curvatus, Carnobacterium divergens, Carnobacterium maltaromaticum* and *Lactococcus lactis* are the main species providing protective cultures for meat/seafood products (Leroi et al., 2015; Comi et al., 2016; Ramaroson et al., 2018; Iacumin et al., 2020), while lactobacilli or even yeast strains can be used for vegetable food biopreservation (Siedler et al., 2019; Truchado et al., 2020; Windholtz et al., 2021). Although several biopreservation technologies are already available on the market, the rate and speed of innovation in this area needs to increase considerably in order to meet global climate-related challenges. Indeed, the scientific literature dealing with biopreservation is considerably larger than the actual application of biopreservation in the food sector. Foods are complex systems because of their diversity, their various physical, chemical, and biological structures, and the numerous processes used to produce them. Moreover, food microbial community dynamics during shelf life depends on abiotic parameters, which are mostly linked to production processes or storage conditions, and biotic parameters where microbial interactions play a major role. The complexity and diversity of food microbial communities has been a major barrier to the widespread use of biopreservation. Therefore, the conception of efficient biopreservation technology requires the implementation of methodological approaches adapted to the high complexity of food ecosystems. Food microbiology has long been studied by classical culture-dependent methods. During the last decade, omics techniques including genomics, transcriptomics, proteomics, metabolomics, culturomics, and phenomics have revolutionised all areas of the life sciences, including food quality and safety assessment, because of their ability to decipher food systems as a whole (Cook and Nightingale, 2018). The application of omics provides a more realistic portrait of the complex interactions occurring in the food ecosystem (Gálvez et al., 2007), and it has significantly increased our understanding of the potential of microbiomes to increase the productivity and sustainability of food systems. These omics approaches have caused a paradigm shift from unsocial undesirable microorganisms colonising food to strongly interacting microorganisms establishing stable networks (Berg et al., 2020). Omics approaches have been applied to explore various aspects of biopreservation, such as selection and characterisation of protective cultures, investigation of the mechanisms involved in the protective effect, or the impact on food microbial communities. After a brief overview of food biopreservation, the different omics approaches used in studies dealing with biopreservation are reviewed below and analysed by illustrating to what extent they can answer questions related to the impact of biopreservation on the food microbial ecosystem.

## Selection of biopreservation agents

Classical approaches to identify protective culture candidates are mainly based on the detection of inhibition zones in laboratory conditions. Recently, the use of phenomics and genomics has proved highly effective. Phenomics can be defined as the high-throughput study of phenotypes. In the case of biopreservation, the phenotype of interest is the inhibition of spoilage microorganisms or food-borne pathogens. Phenomics has been successfully used to identify strains exhibiting remarkable anti-*L. monocytogenes* properties by using a high-throughput liquid handling system and a genetically engineered luminescent strain of *L. monocytogenes* to set up mixed culture competition assays in food matrices (Riedel et al., 2007; El Kheir et al., 2018). This method was first used to select anti-*Listeria* candidates from a collection of strains isolated from raw milk and a collection of *Lactococcus piscium*. The majority of the candidates obtained did not produce an inhibitory halo following a classical agar diffusion-based method, suggesting promising inhibitory mechanisms (El Kheir et al., 2018). Lately, this high-throughput competition assay was implemented to study the inhibition phenotype of a collection of *C. maltaromaticum* strains under multiple varying conditions. This method resulted in the selection of robust antagonistic *C. maltaromaticum* strains whose anti-*Listeria* properties are insensitive to fluctuations, i.e., inoculation level and time lag of *L. monocytogenes* and candidate inoculation (Borges and Revol-Junelles, 2019). It is expected that phenomics approaches extended to other target microorganisms (Besnard et al., 2021), as well as other phenotypes related to the sensory profile or use of nutrients (Wiernasz et al., 2017; Acin-Albiac et al., 2020), may in the future enable the identification of microorganisms with high biopreservation performances.

Genomics is an interesting approach for selecting protective cultures as genome mining may point out important features that can be involved in the preservation effect (Baltz, 2019), and also prove the absence of some unwanted functions such as antibiotic resistance or biogenic amine synthesis. Genome mining involves the analysis of functional gene annotation, resulting in particular from antiSMASH (Blin et al., 2017) and BAGEL (van Heel et al., 2018), which are designed to identify clusters of genes involved in the biosynthesis of antimicrobial compounds, combined with genome comparison to find correlation between the presence/absence of genes and protective properties. As an example, the genome sequence of *L. sakei* 23K, a meat adapted bacterium used as a starter for sausage fermentation, but also proposed as a biopreservative agent for raw meat products (Zagorec and Champomier-Vergès, 2017), revealed its strong ability to be competitive in meat products (Chaillou et al., 2005; Eijsink and Axelsson, 2005). Indeed, genome analysis highlighted the presence in the genome of elements putatively enabling the use of alternative carbon sources, such as ribose, inosine, and adenosine. Their efficacy was subsequently proven and helps to explain the fitness in meat of *L. sakei*, which thereby escapes competition for energy sources (Rimaux et al., 2011). Also, the requirement for haem and iron, two components present in meat, could be assessed by genomics through the gene repertoire and further evidenced by functional genomics (Duhutrel et al., 2010; Verplaetse et al., 2020).

Another example of genomics input is the production of antagonistic molecules by protective strains. This has long been studied through the production of bacteriocins. The genome sequence analysis of *L. curvatus* CRL705, a strain known to produce two bacteriocins (lactocin 705 and AL705), revealed the presence of additional genes putatively involved in bacteriocin production (sakacin P, sakacin Q, sakacin X, and sakacin T; Hebert et al., 2012). Divercin V41 is a bacteriocin involved in the protective function of *C. divergens* V41 a lactic acid bacterium strain whose operon sequence was reported more than two decades ago (Metivier et al., 1998). The genome analysis of this strain revealed that an additional gene was present in the divercin V41 operon (Remenant et al., 2016), the function of which was shown to be important for bacteriocin production (Back et al., 2015). Comparative genomics of *Carnobacterium* highlighted potential new candidate strains for biopreservation, efficient against *L. monocytogenes* and harbouring original bacteriocin gene equipment. For example, five different bacteriocins and a 16 kDa new one were predicted in the *C. maltaromaticum* SF668 and *C. maltaromaticum* EBP3019 genomes, respectively (Begrem et al., 2020). Combining genome analysis and peptidomics of a *Companilactobacillus crustorum* strain enabled, from the peptides produced during the growth of this strain, the discovery of eight novel bacteriocins and two other antimicrobials with a broad spectrum of action against Gram-positive and Gram-negative pathogens (Yi et al., 2018).

*L. piscium* CNCM I-4031 (EU2241) is a protective strain for seafood products that improves the sensory quality of shrimp and cold-smoked salmon (Fall et al., 2012; Leroi et al., 2015). This strain is also particularly efficient against the pathogen *L. monocytogenes* by reducing growth and virulence (Saraoui et al., 2018). Combined phenotyping and genome analyses evidenced that the inhibitory effect is dependent on cell-to-cell contact instead of extracellular molecules such as bacteriocins, organic acids, or hydrogen peroxide (Saraoui et al., 2016; Marché et al., 2017). This unusual mechanism still remains to be elucidated; nevertheless medium- and high-throughput screening revealed that other strains of the same species could be selected as new protective cultures, notably related to their large antimicrobial capacities (Wiernasz et al., 2017; El Kheir et al., 2018).

Genomics can reveal other unexpected features, as exemplified by the genome sequence of *C. divergens* V41 which contains an intriguing long genomic island (∼40 kb) encoding polyketide synthases/non-ribosomal peptide synthases (PKS/NRPS) or PKS/NRPS-like enzymes, putatively involved in the production of a secondary metabolite of unknown function (Remenant et al., 2016). Such molecules may have antimicrobial functions or be associated with oxidative stress resistance, and immunomodulatory or cytotoxicity activities. Comparative genomic analysis of *Carnobacterium* strains showed that this PKS/NRPS gene cluster is unique in *C. divergens* V41 (Begrem et al., 2020). Other PKs/NRPs antimicrobial compounds, such as milkisin produced by a *Pseudomonas* sp. strain, with potentially interesting properties for the biopreservation of milk products have been described (Schlusselhuber et al., 2018, 2020). Reuterin-producing *Limosilactobacillus reuteri* are known as bioprotective agents for dairy products (Ortiz-Rivera et al., 2017). Some rare strains harbour a PKS/NRPS genomic island encoding *rtc* genes involved in the synthesis, regulation, immunity, and secretion of an antimicrobial tetramic acid named reutericyclin (Gänzle et al., 2000; Lin et al., 2015).

While many published papers describe the screening and evaluation of bioprotective antifungal lactic acid bacteria strains *in vitro* and *in situ* (Delavenne et al., 2012; Leyva Salas et al., 2018), as well as the identification of metabolites involved in their antifungal activities, the application of genomics and functional genomics to the selection of antifungal microorganisms is still in its infancy. Studies providing insights into the metabolic pathways of antifungal metabolites such as phenyllactic acid (Wu et al., 2020), clearly point out that in-depth studies of antifungal lactic acid bacteria using comparative and functional genomics are needed. Such studies should help to elucidate yet uncharacterised biosynthetic pathways of known antifungal molecules and potentially reveal new ones and to establish whether other competition exclusion phenomena exist between lactic acid bacteria and spoilage fungi, with the objective to further understand their action mechanism and possibly provide helpful tools for strain selection.

Some species with bioprotective properties are also described as potential spoilage organisms, depending on the type of food or process (Brillet et al., 2004; Leisner et al., 2007; Andreevskaya et al., 2015; Leroi et al., 2015; Saraoui et al., 2016; Poirier et al., 2018). Thus comparative genomics between strains known to be responsible for spoilage or on the contrary known as protective cultures should be an interesting approach for the selection of strains of interest. In addition, as a complementary approach to phenotypic tests, genome analysis of potent protective strains can be a complementary tool for their safety assessment i.e., screening for the presence of genes related to biogenic amine production, antibiotic resistance genes, as well as their location with respect to mobile genetic elements, i.e., plasmids and bacteriophages. Such an approach was recently applied to a *Lactiplantibacillus plantarum* starter culture used in the manufacturing of *nahm* fermented pork (Chokesajjawatee et al., 2020), as well as to a potent *L. plantarum* protective culture (Barbosa et al., 2021).

## Impact of biopreservation on targeted microorganisms

**FIGURE 1:**
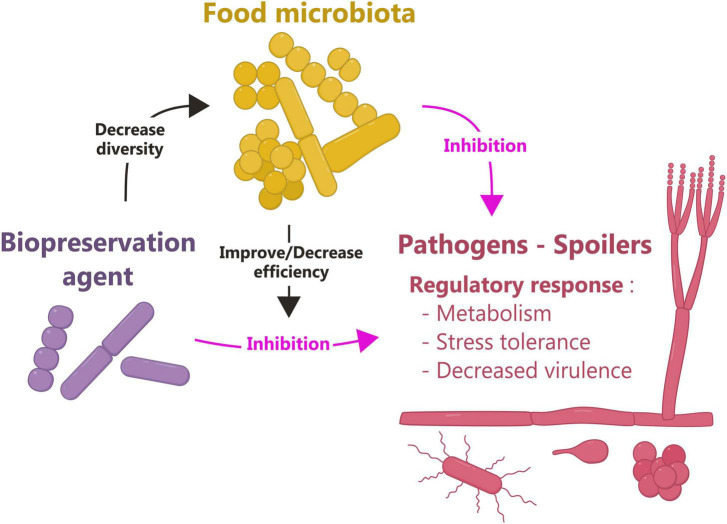
Major advances in the field of biopreservation thanks to omics approaches. The objective of biopreservation is to induce growth cessation or decay of pathogens and spoilers. Omics approaches have shown that in response to biopreservation, target microorganisms modulate the expression of genes involved in cell metabolism, stress tolerance and virulence. In addition, omics revealed that biopreservation can decrease the diversity of food microbiota. Conversely, depending on its structure, the food microbiota can either improve or reduce the effectiveness of biopreservation agents. Food microbiota can also have intrinsic protective properties and thus inhibit unwanted microorganisms.

The role of protective cultures is mainly related to their fitness (nutritional competition, ability to resist harsh conditions encountered in food), enabling them to establish and dominate at the expense of other undesirable species, as well as to their ability to produce antimicrobial molecules, such as organic acids, hydrogen peroxide or specialised metabolites as bacteriocins, biosurfactants, or lipopeptides (Ben Said et al., 2019; Bourdichon et al., 2021; Table 1). The expected impact is induction of decay or at least growth inhibition of the undesirable microorganism. Classical methods such as qRT-PCR are *a priori* methods that involve a prior selection of the target genes to be studied. On the contrary, omics approaches such as RNAseq can be used without *a priori*, opening the possibility to identify original unsuspected mechanisms. These approaches have revealed that the mechanisms involved are more complex than previously thought, and involve microbial sensing capacity, gene regulation, and the potential for combining antimicrobial compounds for microbial inactivation (Figure 1).

**TABLE 1:** Main characteristics of biopreservation in food and mechanisms involved.

Protective mode of action	Resulting effect	Effect at cellular and/or molecular levels	Mode of use	References
Nutritional competition	- Jameson effect - Growth impairment because of lack of nutrients	- Early entry into stationary phase of targeted microorganisms, and protective cultures - Growth cessation of targeted microorganisms	Live microorganisms added to the food	Jameson, 1962; Guillier et al., 2008; Hibbing et al., 2010
Production of organic acids	- Extracellular pH drop - Diffusion across the microbial cytoplasmic membrane	- Cytoplasmic pH decrease - Collapse of the proton gradient across the membrane - Disruption of cellular processes		Warnecke and Gill, 2005
Production of hydrogen peroxide	- Oxidation of cellular components	- Peroxidation and disruption of membrane layers - Oxidation of oxygen scavengers - Enzyme inhibition - Oxidation of nucleosides - Disruption of protein synthesis - Growth decrease at low concentrations - Cell death at high concentrations		Finnegan et al., 2010
Respiration	Change in atmosphere composition (O_2_ decrease) leading to microaerophilic conditions	- Inhibition of strict aerobic bacteria	Live microorganisms added to the food	Ben Said et al., 2019
Production of bacteriocins	Bactericidal or bacteriostatic activity against species taxonomically related to the producing strain	- Pore formation in the cytoplasmic membrane - Loss of structure and subsequent cell death	Live microorganisms added to the food	Elsser-Gravesen and Elsser-Gravesen, 2013
			Bacteriocins purified from cultivated producer strains	Martinez et al., 2016

### Listeria monocytogenes

Two recent studies have explored the mechanism of inhibition of *L. monocytogenes* by antimicrobial lipopeptide (P34) or the peptide nisin (encapsulated or not) by proteomic analysis after incubation in a laboratory medium (Pinilla et al., 2021; Stincone et al., 2021). The lipopeptide P34 caused the downregulation of proteins involved in manganese transport and upregulation of proteins related to iron transport in *L. monocytogenes*. In addition, reduction of stress tolerance proteins related to the sigma B and VirR regulons and modulation of phosphoenolpyruvate phosphotransferase systems for sugar transport were observed (Stincone et al., 2021). Exposure of *L. monocytogenes* to nisin induced the synthesis of proteins related to ATP-binding cassette transporter systems, transmembrane proteins, RNA-binding proteins, and diverse stress response proteins (Pinilla et al., 2021). Some of the proteins detected in the presence of free nisin were related to translocation of *L. monocytogenes* virulence factors, activation of the LiaR-mediated stress defence, and glycosylation of cell wall teichoic acid. The comparison of treatment by free and encapsulated nisin revealed that *L. monocytogenes* did not express some stress proteins when nisin was encapsulated, suggesting the production of nisin-resistance factors by exposure to encapsulated nisin. The authors suggested that liposomes allow controlled release of nisin in the medium, resulting in fewer interactions between nisin and bacteria compared with free nisin, which may impact the mechanism of action of nisin (Pinilla et al., 2021). The induction of resistance factors was also observed by exposing *L. monocytogenes* to sublethal doses of pediocin. Transcriptomic analysis revealed the expression modulation of the two-component system LisRK and the alternative sigma factors SigB and SigL, resulting in an increased resistance of *L. monocytogenes* to this bacteriocin (Laursen et al., 2015).

Recently, the ability of *C. maltaromaticum*, *Leuconostoc gelidum*, or *L. piscium* strains to inhibit *L. monocytogenes* was demonstrated in seafood (Saraoui et al., 2016, 2018; Wiernasz et al., 2017). *L. piscium* inhibition required cell-to-cell contact with *L. monocytogenes*, affecting its cell surface, and decreasing its virulence (Saraoui et al., 2018). The metabolomic fingerprints suggested that this inhibition might not involve nutritional competition and remains to be explored (Saraoui et al., 2016).

### Staphylococcus aureus

*Lactococcus garvieae* was shown to inhibit *S. aureus* growth in milk, in cheese, and *in vitro*, potentially through hydrogen peroxide (H_2_O_2_) production (Delbes-Paus et al., 2010). To better characterise this mechanism of inhibition *in vitro*, the transcriptomes of *L. garvieae* and *S. aureus* co-culture have been explored by RNA-seq and RT-qPCR (Delpech et al., 2015, 2017). *L. garvieae* repressed the expression of *S. aureus* genes involved in stress response, including oxidative stress generated by H_2_O_2_, and in cell division. It also modulated the expression of virulence-related genes (particularly *agrA*, *hld*, and enterotoxin-encoding genes; Delpech et al., 2015). For *L. garvieae*, a high concentration of H_2_O_2_ was not associated with higher expression of the H_2_O_2_ synthesis genes *pox*, *sodA*, and *spxA1*, but rather with repression of H_2_O_2_-degradation genes (*trxB1*, *ahpC*, *ahpF*, and *gpx*; Delpech et al., 2017). The interaction between *L. lactis* and *S. aureus* has also been widely studied and transcriptomic analyses were performed with microarrays and RT-qPCR. In a chemically defined medium held at a constant pH value of 6.6, the growth of the two bacteria in co-culture was not modified, but their transcriptome was modulated (Even et al., 2009; Nouaille et al., 2009). The expression of *S. aureus* virulence-related genes was impaired by *L. lactis*: the expression of genes encoding global regulators, including *agr* and consequently the *agr*-controlled enterotoxin genes and *sar*, was strongly reduced (Even et al., 2009). This downregulation of *agr* in *S. aureus* was associated with the reducing properties of *L. lactis* (Nouaille et al., 2014). *L. lactis* genes associated with amino acid metabolism, ion transport, oxygen response, menaquinone metabolism, cell surface, and phage expression were differentially expressed in co-culture compared to monoculture (Nouaille et al., 2009). In a complex medium such as the cheese matrix, the acidifying, proteolytic, and reducing activities of *L. lactis* were shown to affect carbohydrate and nitrogen metabolisms and the stress response of *S. aureus* (Cretenet et al., 2011). Enterotoxin gene expression was positively or negatively modulated by both *L. lactis* and the cheese matrix itself, depending on the enterotoxin type. Again, the *agr* operon was downregulated by the presence of *L. lactis*, in part because of a drop in pH (Cretenet et al., 2011). All these data highlight the intimate link between environment, metabolism, and virulence expression. A third binary interaction between *Enterococcus faecalis* and *S. aureus* was studied in milk and in cheese (Viçosa et al., 2018; Nogueira Viçosa et al., 2019). When co-cultured, the growth of *S. aureus* was decreased and the classical enterotoxins were not produced. The expression of several enterotoxins and global regulator genes (including *agr*) was downregulated, while the expression of genes involved in metabolism was upregulated. Finally, the interaction of *S. aureus* with a mixed culture of *Enterococcus durans, E. faecalis* and *L. lactis* in milk confirmed that the production of enterotoxins was reduced in mixed culture and the expression of several genes involved in virulence was inhibited (Zdenkova et al., 2016). All these studies on the interaction of *S. aureus* with different lactic acid bacteria converge on a very important idea that the mechanism of action of biopreservation can result in the inhibition of virulence by inhibiting the production of enterotoxins through the decreased expression of genes involved in their synthesis.

### Pathogenic *Escherichia coli*

*L. curvatus*, *L. plantarum*, and *Enterococcus mundtii* strains can inhibit the growth of *E. coli* O157:H7 when co-cultured in a meat model medium (Orihuel et al., 2018). The antagonistic effect of the most efficient *E. mundtii* strains against *E. coli* O157:H7 were characterised by a proteomic analysis (Orihuel et al., 2018). The expression of *E. mundtii* proteins involved in carbohydrate/amino acid metabolisms, energy production, transcription/translation, and cell division was modified in the presence of *E. coli*. Reciprocally, the presence of *E. mundtii* resulted in repression of *E. coli* synthesis of proteins related to metabolism and transport of amino acids and nucleotides, as well as overexpression of proteins involved in stress, energy production, and transcription (Orihuel et al., 2019). In addition, proteins associated with adhesion to extracellular matrix proteins of meat were modulated in *E. coli* in accordance with its decreased adhesion capacity when co-cultured with *E. mundtii. E. mundtii* did not influence the lytic cycle of the *E. coli* O157:H7 strain, indicating its potentially safe use as a bioprotective agent, since engagement in the lytic cycle results in the production of shiga toxin (Orihuel et al., 2019). The interaction of *E. coli* O157:H7 with ground beef microbiota was also studied (Galia et al., 2017). A beef piece was divided in two parts with the inner part considered sterile and the outer part as encompassing a natural meat microbiota. The microbial community structure was assessed by 16S rDNA amplicon sequencing, and the transcriptome of two inoculated strains (*E. coli* O157:H7 and *E. coli* O26:H11) was studied by RNAseq comparing samples of sterile and naturally contaminated meat. This study revealed that the two *E. coli* strains behave differently. On the one hand, an upregulation of genes involved in detoxification and stress response and a downregulation of *peR*, a gene negatively associated with virulence phenotype, were observed in *E. coli* O157:H7. On the other hand, the interaction of *E. coli* O26:H11 with ground beef microbiota revealed that genes involved in division, peptidoglycan synthesis, DNA repair, metal acquisition, and carbohydrate and amino acid metabolism were downregulated (Galia et al., 2017).

### Fungal food spoilers

In a recent review of biopreservation against moulds in dairy products (Shi and Maktabdar, 2022), the authors point out newly described antifungal mechanisms. Among these is a perfect example of how omics technologies shed new light on the understanding of the protective mechanisms of antifungal lactobacilli toward dairy product spoilage fungi (Siedler et al., 2019). This work revealed that manganese scavenging by *Lacticaseibacillus rhamnosus* and *Lacticaseibacillus paracasei* antifungal strains, previously known as a defence mechanism against oxidative stress, was a main inhibitory mechanism (i.e., competitive exclusion) against many yeast and mould species involved in dairy product spoilage. Indeed, following milk fermentation and supplementation with manganese, their bioactivity was completely lost. A transcriptomic approach based on RNA-seq further showed that one of the most highly expressed gene products in these strains encoded a manganese transporter (MntH1). The role of MntH1 in manganese scavenging was confirmed in a Δ*mntH1 L. paracasei* strain in which no significant antifungal activity was detected, while bioactivity was restored in the Δ*mntH1* mutant complemented with a plasmid containing the *mntH1* gene under its own promoter.

Besides the above-mentioned discovery that competitive exclusion for manganese was an important antifungal mechanism, production of antifungal metabolites and pH decrease were believed to be the main mechanisms involved in the bioactivity of antifungal protective lactic acid bacteria. Through the use of metabolomic targeted and untargeted approaches with or without prior medium fractionation and bioactivity testing, more than 60 molecules have been thought to play a role in antifungal activity (see recent reviews by Leyva Salas et al., 2017 and Siedler et al., 2019). These metabolites include molecules produced through carbohydrate metabolism (e.g., organic acids such as lactic, acetic, formic, and succinic acids, volatile compounds such as diacetyl), proteolysis (e.g., bioactive peptides resulting from casein cleavage), amino acid metabolism (e.g., phenyllactic acid), lipolysis and free fatty acid metabolism (e.g., 3-hydroxydecanoic, caproic – i.e., hexanoic- and caprylic – i.e., octanoic – acids), but also complex compounds derived from bioconversions (e.g., benzoic acid) or peptide synthesis [e.g., cyclic dipeptides such as cyclo(L-Phe-L-Pro) and cyclo(L-Phe-trans-4-OH-L-Pro)] (Ström et al., 2002; Aunsbjerg et al., 2015; McNair et al., 2018; Leyva Salas et al., 2019; Garnier et al., 2020; Shi and Knøchel, 2021a,b). It should be underlined that with the exception of lactic and acetic acids which are produced in g/kg or g/L amounts, these molecules are produced in quite low quantities, all of which are at concentrations far from their individual minimum inhibitory concentration (MIC), thus suggesting that they act in synergy or by additive effects. In a recent study, Leyva Salas et al. (2019) used a metabolomics approach coupled with supervised multivariate analysis to investigate 56 antifungal compounds as well as volatiles. It was found that 9 key compounds including acetic acid, 5 aromatic acids, and three volatiles were associated with antifungal activity against *Mucor racemosus* and *Penicillium commune*, although their concentrations were below their respective MICs. Further investigation on *Penicillium roqueforti* and *Mucor circinelloides* revealed that several combinations presented additive (e.g., diacetyl + 3-phenylpropanoic acid, diacetyl + acetic acid) or synergistic effects (diacetyl + octanoic acid, octanoic + 3-phenyl propanoic acids), clearly reinforcing the idea that additive and synergistic effects of antifungal molecules are involved in lactic acid bacteria bioactivity. To go further, future work could include the investigation of more complex mixtures of antifungal compounds at concentrations close to those encountered in biopreserved foods. Moreover, it is not clear how antifungal molecule synthesis and competitive exclusion interact together in different fungal species with various susceptibilities to protective strains.

## Biopreservation at the microbiome level

The primary objective of biopreservation is to limit the presence of unwanted microorganisms in food. However, the addition of biopreservatives can have an overall impact on the food microbiome. Omics approaches have helped clarify the complex interactions occurring in the food ecosystem and their impact on the organoleptic properties of foods (Figure 1).

### Metagenomics

Prior to the availability of high-throughput DNA sequencing (HTS) techniques, DNA fingerprinting techniques as PCR-denaturing gradient gel electrophoresis or PCR-temporal temperature gradient electrophoresis, targeting mainly the V3 region of 16S rDNA, were employed to determine whether bioprotective lactic acid bacteria strains were able to colonise the food matrix and dominate the microbiota (Hu et al., 2008; Saraoui et al., 2017; Zhang et al., 2018). However, discrepancies between these DNA fingerprinting approaches and cultural methods (Ercolini et al., 2010) have highlighted the need for methods of higher resolution. There are two commonly used HTS methods in microbiome research: amplicon sequencing and metagenomic sequencing (Liu et al., 2021). Amplicon sequencing is the most widely used, including in the field of biopreservation. It demonstrated that the three protective cultures *L. rhamnosus* LRH05, *L. sakei* LSK04, and *C. maltaromaticum* CNB06, alone or in combination, were able to colonise cheese and became dominant after storage (Bassi et al., 2020). In shrimp, the results of amplicon sequencing were consistent with the successful colonisation of the food matrix by the biopreservative strains *L. plantarum* and *Lacticaseibacillus casei*, and a reduction in the relative abundance of *Shewanella*, which includes the spoilage species *Shewanella baltica* (Li et al., 2019). In addition, in beef burgers, the analysis of predicted metagenomes revealed that nisin-activated packaging resulted in a reduction in the abundance of specific metabolic pathways related to spoilage (Ferrocino et al., 2015). Table 2 summarises the different studies using omics approaches to assess the impact of biopreservation on food ecosystems and the main findings.

**TABLE 2:** Overview of omics approaches used to assess the impact of biopreservation on food ecosystems and main findings.

Omics approach	Methodological details	Food	Main finding	References
PCR-Denaturing gradient gel electrophoresis/Temporal temperature gradient electrophoresis associated or not with band sequencing	V3 region of 16S rDNA	Cooked and peeled shrimp	*Carnobacterium divergens* V41 but not *Lactococcus piscium* CNCM I-4031 used to inoculate shrimp dominated and was associated with reduction of off-flavours	Saraoui et al., 2017
	V3 region of 16S rDNA	Vacuum-packaged beef meat	Bands associated with spoilage bacteria (*Pseudomonas*, Enterobacteriaceae, *Brochothrix. thermosphacta*), but not with LAB, disappeared in samples inoculated with *Latilactobacillus sakei* and *Latilactobacillus curvatus* bioprotective strains	Zhang et al., 2018
	V3 region of 16S rDNA	Vacuum-packed cooked ham	Predominant spoilage LAB were not detected when the bioprotective *Latilactobacillus sakei* B-2 strain was used	Hu et al., 2008
	V6–V8 region of 16S rDNA	Beef cuts packaged in nisin-coated plastic bags	Similar diversity in control and nisin-treated samples although differences were observed with plate counts for *Brochothrix thermosphacta*	Ercolini et al., 2010
DNA sequencing	Pyrosequencing of V3–V4 region of 16S rDNA	Cold-smoked salmon	Different OTU ratios were observed between control and samples inoculated with *Lactococcus piscium* EU2241 (= CNCM I-4031). No correlation with sensory analysis	Leroi et al., 2015
	Illumina sequencing of V3–V4 region of 16S rDNA	Raw/peeled shrimp	*Shewanella baltica* significantly inhibited after co-inoculation with *Lactiplantibacillus plantarum* AB-1 and *Lacticaseibacillus casei* LC	Li et al., 2019
	Illumina sequencing of V3–V4 region of 16S rDNA	St Nectaire-type cheese	Implantation of an inhibitory consortium whose inhibitory activity toward *Escherichia coli* O26:H11 depended on indigenous microbiota composition	Frétin et al., 2020
	Illumina sequencing of V3–V4 region of 16S rDNA and of an internal 280 bp fragment of the *gyr*B gene	Diced cooked ham	Bioprotective activity and implantation of a nisin-producing strain of *Lactococcus lactis* depended on microbiota composition	Chaillou et al., 2022
	Illumina sequencing of V4 region of 16S rDNA	Fresh filled pasta	Cultures of *Lactiplantibacillus plantarum* and *Lacticaseibacillus paracasei* were not dominant but reduced the initial microbiota and gave a competitive advantage to other LAB species	Tabanelli et al., 2020
16S rRNA sequencing	Sequencing of V3–V4 region of 16S rRNA from cDNA	Fermented sausage	Large domination of *Lactobacillaceae* and reduction of bacterial diversity in samples inoculated with protective *Latilactobacillus curvatus* strain	Giello et al., 2018
	Sequencing of V3–V4 region of 16S rRNA from cDNA	Beef burgers in nisin-activated packaging	Lower abundance of some taxa in samples with nisin-activated packaging	Ferrocino et al., 2015
Volatilome analysis	Headspace SPME/GC-MS	Cooked and peeled tropical shrimp	Inhibition of *Brochothrix thermosphacta* by *Lactococcus piscium* CNCM I-4031 correlated with attenuation of off-odours and diminution of some volatile compounds	Fall et al., 2012
	Headspace SPME/GC-MS	Salmon gravlax	6 protective strains exhibited their own volatilome profiles. Quality improvement was not correlated with implantation of protective culture	Wiernasz et al., 2020
	Headspace SPME/GC-MS	Fresh filled pasta	*Lacticaseibacillus rhamnosus* and *Lacticaseibacillus paracasei* influenced the aroma profile with overall acceptability of the product	Tabanelli et al., 2020
	NMR spectroscopy	*in vitro*	Kinetic analysis of 11 major metabolites involved in the metabolism of *Lacticaseibacillus rhamnosus* and *Lacticaseibacillus plantarum*	Ebrahimi et al., 2016
	FTICR-MS	Red wines	No effect on the volatile compounds of a *Metschnikowia pulcherrima* bioprotective strain. Wines produced from bioprotected or sulphited must had different metabolic signatures	Simonin et al., 2020

HTS approaches open up the possibility of going much further than simply answering the question of the implantation success of the protective microorganisms and their effect on the targets. Amplicon sequencing can be used to estimate the impact of biopreservation on food microbiota. When batches of cold-smoked salmon were inoculated with a bioprotective strain of *L. piscium*, the microbiota structure differed significantly between control and biopreserved products after 3 weeks of storage (Leroi et al., 2015). The impact of biopreservation strains can be stronger, as in the case of fermented sausages where 16S rRNA-based analysis revealed a markedly lower microbial diversity of the metabolically active microbial community (Giello et al., 2018). Thus, although special attention is paid to the selection of strains with a narrow antimicrobial spectrum in biopreservation (e.g., bacteriocin-producing bacteria), biopreservation can end up with significant changes in food microbiota structure. Although this side effect can be undesirable, especially in fermented foods, in some circumstances it is possible to take advantage of this broad impact on food microbiota to target spoilage microorganisms, which can encompass a large number of phylogenetic taxa. In dairy products such as low-salt fresh cheeses, spoilage bacteria are mainly psychrotrophic Gram-negative species, including several *Enterobacteriaceae* and *Pseudomonas* spp. (Ledenbach and Marshall, 2009; Spanu et al., 2018; Bassi et al., 2020). The three lactic acid bacterial strains *L. rhamnosus* LRH05, *L. sakei* LSK04, and *C. maltaromaticum* CNB06, added alone or in combination, were found after 5 weeks of storage to be effective in inhibiting the Gram-negative bacteria population of fresh Primo Sale cheese inoculated with a cocktail of 10 bacterial spoilage isolates (Bassi et al., 2020). In Italian fresh filled pasta cheese, the impact of protective cultures at the community level can be used to reduce the initial microbiota associated with raw materials and to confer a competitive advantage on safer or more acceptable bacterial species such as *Leuconostoc mesenteroides*, at the expense of more problematic species such as *Streptococcus uberis* and *Streptococcus parauberis* (Tabanelli et al., 2020).

While, on the one hand, biopreservation can have an impact on the microbiota, on the other hand, the microbiota can also affect the efficiency of biopreservation. The protective property of a nisin-producing *L. lactis* strain was tested in combination with high pressure under controlled conditions of microbiota composition in reduced-nitrite diced cooked ham (Chaillou et al., 2022). Sterile diced ham cubes were inoculated with two different microbiota collected from cooked hams, together with the protective *L. lactis* strain prior to vacuum packaging and high-pressure treatment. During storage, the two selected cooked ham microbiota were both characterised by a microbial community enriched in high potential spoilage bacterial species (especially *Proteobacteria*). Comparison of the bacterial community composition after 1 month of storage revealed that the protective effect of the *L. lactis/*high-pressure combination is highly dependent on the ham microbiota. Indeed, when ham samples were inoculated with *Pseudomonas* spp. and *Serratia* spp. rich-microbiota, *L. lactis* became dominant (>90% relative abundance). However, when ham samples were instead inoculated with microbiota dominated by *Psychrobacter* sp. and *Vibrio* sp., *L. lactis* was not competitive and *Brochothrix thermosphacta* became dominant (Chaillou et al., 2022). Thus, by interacting with the protective strain, food microbiota can act on the efficiency of biopreservation. By contrast, it is also possible that the microbiota can contribute to biopreservation in concert with the protective strains by acting directly on the unwanted microorganism through its intrinsic protective properties. In uncooked pressed cheese, the protective activity of a consortium comprising three strains belonging to the species *H. alvei, L. plantarum*, and *L. lactis* was dependent on the composition of the microbiota colonising the processed raw milk. The raw milk batches associated with the lowest growth of *E. coli* O26:H11 were characterised by greater relative abundance of lactic acid bacteria, the three *Gammaproteobacteria Acinetobacter, Serratia*, and *Hafnia*, as well as *Macrococcus*. On the other hand, the highest levels of *E. coli* O26:H11 were observed when the milk microbiota was significantly enriched with bacteria from the genera *Ramboutsia, Paeniclostridium*, and *Turicibacter* (Frétin et al., 2020).

Overall, these data were mainly produced thanks to amplicon sequencing. The major drawbacks of this approach are the biases in relative abundances resulting from PCR amplification and differences in the number of ribosomal operons between species (Edgar, 2017), the limited taxonomic resolution, mainly at the genus level even if some efforts have been made in developing specialised databases such as the DAIRYdb (Meola et al., 2019), and the lack of functional information. Amplicon sequencing targeting housekeeping genes such as *gyrB* is also promising for a better identification at the species or even intra-species level, with also less bias for relative abundance determination (Poirier et al., 2018). Investigating food communities by using shotgun metagenomics could help to improve accuracy, especially by gathering information at the species or even the strain level, and could give a global view of the functions involved in the process of biopreservation at the microbial community scale. Furthermore, network analysis of metagenetic and metagenomic data could be used to identify patterns (i.e., co-occurrence and mutual exclusion) in food microbial communities, biopreserved or not. Such an approach would enable hypotheses to be drawn regarding biotic interactions occurring between microorganisms, which could then be tested experimentally, and could be applied to the selection of potential candidates for biopreservation or for a deeper understanding of the impact of selected bioprotective cultures at a community level.

### Metabolomics

In addition to their impact on food microorganisms, biopreservation agents are likely to modify the properties of the food matrix and in particular its organoleptic characteristics. Metabolomics approaches allow us to go much further than sensory analyses in the study of the impact of biopreservation agents on the matrix. SPME/GC-MS showed that cooked peeled shrimp contains a reduced amount of unwanted aldehydes and alcohols associated with sensory spoilage when *L. piscium* is used as an inhibitor of *B. thermosphacta* (Fall et al., 2012). Biopreservatives can thus play a positive sensory role by inhibiting a target microorganism responsible for spoilage. However, biopreservation can also negatively impact the matrix and can be responsible for significant changes, highlighting the interest of the polyphasic omics approach to the selection of candidate strains. An exemplary study describes the use of Head Space-Solid Phase MicroExtraction/Gas Chromatography- Mass Spectrometry (HS-SPME/GC-MS) to reveal that the impact of biopreservation candidates varied dramatically depending on the strain considered, and that specific signatures could be associated with each strain. Coupled to microbial community structure investigation by amplicon sequencing, the authors were able to rationally select two strains with the highest protective effect and the lowest sensory activity in salmon gravlax (Wiernasz et al., 2020; Table 2). Nuclear Magnetic Resonance (NMR) also has great potential in the study of living organisms, owing to its non-destructive nature, i.e., it can be used for *in vivo* and *in vitro* measurements of biological processes, with no quenching of the metabolism required. NMR spectroscopy can be particularly helpful for the kinetic analysis of the metabolism of protective cultures, as in the development of *in vitro* NMR kinetic measurements of lactic acid bacteria (Ebrahimi et al., 2016; Table 2). Such a polyphasic approach was also used successfully in wine to assess biopreservation as an alternative to sulphites (Simonin et al., 2020). The wine metabolome being of high complexity, an ultra-high-resolution mass spectrometric method -the Fourier transform ion cyclotron resonance mass spectrometry- was used to identify and annotate more than 7,000 molecules. Clustering analysis revealed that even if the biopreservation agent has a molecular impact on the product, it is significantly lower than the winery effect, showing that biopreservation has preserved the typicality of the products, which can be of high relevance for products with protected designation of origin (Simonin et al., 2020; Table 2).

### Limits of global omics in structured food matrices

Genomic tools based on amplicon sequencing, although powerful for food microbial ecosystem description, may show some limits for biopreserved products. Bioprotective cultures inoculated at a high level are usually dominant, at least at the beginning of storage. As it is generally admitted that taxa representing less than 0.01% of the dominant ones are not detected with metagenetics, the richness of biopreserved food may be underestimated. In the gut, the use of shotgun metagenomics has revealed that the majority of species harbour multiple strains (Ellegaard and Engel, 2016). For instance, despite a low species complexity, the infant gut microbiota exhibits extensive intra-species diversity, with an average of 4.9 strains per subject (Luo et al., 2015). In food, low resolution is a limitation in estimating the impact of protective cultures on subdominant species, such as pathogens usually present at low levels (<10^2^ CFU/g). This problem is less important for specific spoilage organisms that generally dominate at sensory rejection time. However, the interaction between bacteria can modify the sensory characteristics (Stohr et al., 2001; Joffraud et al., 2006; Silbande et al., 2018), and it may be that underestimated taxa play a role in the deterioration of food. In addition, most efforts have been focused on bacteria, and to a lesser extent on yeasts and moulds, although phages, viruses, or *Archaea* may also be present in food microbiota (Roh et al., 2010; Park et al., 2011).

Omics are powerful tools in analysing protective cultures and their interactions at the population level. However, most of these techniques hardly consider microbial population heterogeneity in food systems that can trigger important community functions. Diversification of cell types can originate from genetic variation, ageing, gene expression stochasticity, and environmental condition fluctuation (Bury-Moné and Sclavi, 2017). In structured food matrices such as ground meat or cheese, microorganism microenvironments are highly heterogeneous and vary over time depending upon local microbial activities (Ferrier et al., 2013; Jeanson et al., 2015). Above critical textural levels, individual cells are not able to sediment or swim inside the matrix and eventually grow as large 3D microcolonies (Darsonval et al., 2021; Saint Martin et al., 2022). Microcolony size and sphericity depend on several factors such as local rheological properties, nutrient availability, competing microbiota, and associated interference interactions (Verheyen et al., 2018). Sharp gradients of nutrients and metabolites are generated inside and around the colonies, thus expanding the functional diversification of the local populations. In other structured biosystems such as bacterial biofilms (Lenz et al., 2008; Pérez-Osorio and Franklin, 2008) or gut (Consentino et al., 2021), laser capture microdissection has been put to use in applying omics to defined spatial regions or localised subpopulations of the sample. This could be of interest in deciphering the local behaviour of protective cultures and their targets in distinct and heterogeneous regions of food matrices. The few studies that have approached population functional heterogeneity in real food matrices took advantage of fluorescence microscopy associated with strains reporting the expression of genes of interest by fluorescent proteins (Fleurot et al., 2014; Hernández-Galán et al., 2017). However, food matrices are often opaque to fluorescence microscopy and the density of the population of interest can be too low for reliable quantitative measurements (e.g., bacterial pathogens < 10^2^ CFU/g). Synthetic microbial ecology approaches, where the complexity of the communities and the factors of influence are reduced to their minimum, but are increased in their controllability, can be used to examine interactions and ecological theories (Connell et al., 2012, 2014; Rothschild, 2016; Hynes et al., 2018). Such approaches can be combined with new creative experimental designs for the study of previously unexplored aspects of bacterial behaviour in spatially structured populations (Connell et al., 2013; Wessel et al., 2013; Bridier et al., 2017). In particular, 3D bioprinting of simplified structured matrices with patterned microbial ecosystems could help study population heterogeneities and interspecies interactions at a single-cell scale in structured matrices (Moon et al., 2016; Kyle, 2018; Gyimah et al., 2021; Krishna Kumar et al., 2021). Target microorganisms could be fluorescently tagged for their geolocalisation and the feeding of spatial models of interactions (with food components and with other microorganisms during growth in the printed matrix; Krishna Kumar et al., 2021). Recently, a transcriptome-imaging approach (par-seqFISH for *parallel sequential fluorescence in situ hybridisation*) was reported to capture gene expression and spatial context within microscale assemblies at a single-cell and molecule resolution and could be put to use in such synthetic ecology approaches (Dar et al., 2021). Biopreservation studies could also benefit from microfluidic approaches to assess small-scale interactions between microorganisms (Burmeister and Grünberger, 2020).

The consideration of population heterogeneity in structured food matrices is starting to be integrated into mathematical spatial modelling and predictive microbiology (Verheyen and Van Impe, 2021). Predictive microbiology is useful to quantify both the impact of biopreservation on the food matrix and to simulate the behaviour (survival, growth, inactivation) of the undesirable target microorganism (Leroy and De Vuyst, 2003; Koutsoumanis et al., 2004; Couvert et al., 2010; Habimana et al., 2011; Møller et al., 2013). Another important point to consider is that the contamination of food products with pathogens such as *L. monocytogenes* and *E. coli* O157:H7 occurs accidentally and usually at low levels, thus requiring single-cell level approaches. Individual-based modelling combined with microenvironment (pH, aw variabilities) modelling of vacuum-packed cold-smoked salmon was more effective in describing variability in the growth of a few *L. monocytogenes* cells than the traditional population models (Ferrier et al., 2013; Augustin et al., 2015). Such stochastic approaches need to be improved by characterising a wider range of microenvironmental factors, such as the variability of the viscosity within food matrices between liquid and solid states, as well as considering biotic factors, namely food components and food microbial communities. However, they could provide complementary information about the behaviour of unwanted microorganisms at realistic contamination levels.

## Conclusion

Omics tools have become essential in the field of biopreservation, both for selecting innovative agents and for studying their effectiveness, their mechanism of action, and their impact on the food ecosystem. These approaches have revealed the diversity and complexity of the molecular mechanisms responsible for the protective activity of biopreservation agents. They have provided fundamental knowledge about biopreservation issues in terms of community description (taxonomy), biotic interactions, and impact on the organoleptic quality of the product. They have also shown that the food microbiota plays a major role in biopreservation by acting positively or negatively (Figure 1). It is now clear that the food microbiota must, in the future, be fully integrated into the biopreservation system engineering process to bring the field into the dimension of food microbiome engineering, so that it can play its protective role against pathogens and spoilage microorganisms as well as serve its technological purpose. Moreover, beyond the functioning of the food microbiome, this engineering process must better integrate its interconnections with other microbiomes (soil, water, plant, animal and consumer) to avoid disrupting their functioning and even to contribute to their balance. In this respect, efforts should be pursued to make more extensive use of multi-omics approaches and to combine them with other complementary approaches that take into account the heterogeneity of microorganisms at the cellular, population, and community levels, as well as the heterogeneity of the food matrix.

## Author contributions

FB and MZ coordinated the work and consolidated the manuscript. All authors contributed to the design of the review, carried out the bibliographic data search, drafted the manuscript and approved the submitted version.
